# Respiratory bellows-gated left atrial late gadolinium enhancement

**DOI:** 10.1186/1532-429X-13-S1-P262

**Published:** 2011-02-02

**Authors:** Dana C Peters, Benjamin R Knowles, Mehdi Hedjazi Moghari, Reza Nezafat, Warren J Manning

**Affiliations:** 1 Beth Israel Deaconess Medical Center, Boston, MA, USA

## Purpose

To compare left atrial (LA) late gadolinium enhancement (LGE) imaging using bellows- and NAV-gating.

## Introduction

The LGE method can visualize post-ablation scar in the LA, using increased spatial resolution (1-2). However, the inversion pulse required for LGE also nulls the liver signal at the time of data acquisition (optimal TI time), so that NAV-gating, used for respiratory compensation, becomes impossible. The NAV-restore (3), which reinverts the region of the liver immediately after the initial inversion pulse, permits gating. However, pulmonary vein (PV) blood magnetization is also reinverted, and flows into the left atrium, generating inflow artifacts. These artifacts have been addressed by 1) a NAV with a smaller diameter, 2) monitoring applied earlier before data acquisition (4), or directly after data acquisition (5), when the liver magnetization will be non-zero due to a shorter or longer effective TI (with no NAV restore needed). However, optimal NAV-gating demands monitoring coincident with acquisition of central k-space. Bellows-gating is an early respiratory compensation method, with recent data suggesting a strong correlation with the superior-inferior diaphragmatic motion (6-9). Here we present the results of bellows-gated LGE.

## Methods

Nine healthy subjects (5F, age = 26±14) were imaged in random order 15-25 minutes post contrast with identical 3D LGE sequences, using NAV-gating (5mm window with no tracking, with NAV prior to data acquisition) and commercially provided bellows-gating. The LA was imaged using an axial (N=6), or LV short-axis (N=3) orientation with parameters as previously described (1). Image sharpness and ghosting were evaluated on scale of 1-4 (4=excellent, 3=good, 2=fair, 1=poor); the appearance of inflow artifacts in the right PVs were noted (0=none, 1=moderate, 2=severe).

## Results

**Figure 1:**
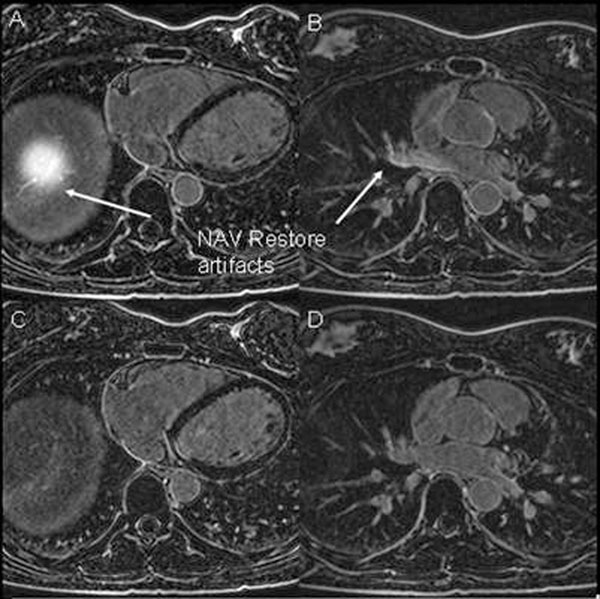
A, B) NAV-gated LA LGE, showing the NAV-restore artifacts on the liver (A), and in the right superior PV (B). C,D) Bellows gated LA LGE, with similar quality.

Figure 1 compares bellows and NAV-gating. The average image sharpness was 3.0±0.5 vs. 2.7±1.0, with average ghosting of 3.1±0.8 vs. 3.1±0.6, for NAV and bellows-gating respectively (p=NS). For NAV-gating, the inflow artifacts affected the right superior PV, with an average grade of 1.7±0.4 (severe), and the right inferior PV, with a grade of 0.7±0.4 (moderate).

## Conclusions

For 3D LGE LA imaging bellows-gating can provide similar respiratory compensation as the NAV, without inflow artifacts.
